# Alpha-Defensin versus Leukocyte Esterase in Periprosthetic Joint Infection: An Updated Meta-Analysis

**DOI:** 10.1155/2020/3704285

**Published:** 2020-11-18

**Authors:** Zhizhuo Li, Qingyu Zhang, Lijun Shi, Fuqiang Gao, Wei Sun, Zirong Li

**Affiliations:** ^1^Department of Orthopedics, Peking University China-Japan Friendship School of Clinical Medicine, 2 Yinghuadong Road, Chaoyang District, Beijing 100029, China; ^2^Department of Orthopedics, Shandong Provincial Hospital Affiliated to Shandong University, No. 324, Road Jing Wu Wei Qi, Jinan, 250021 Shandong, China; ^3^Department of Orthopedics, Graduate School of Peking Union Medical College, China-Japan Friendship Institute of Clinical Medicine, 2 Yinghuadong Road, Chaoyang District, Beijing 100029, China; ^4^Beijing Key Laboratory of Immune Inflammatory Disease, China-Japan Friendship Hospital, 2 Yinghuadong Road, Chaoyang District, Beijing 100029, China

## Abstract

Periprosthetic joint infection (PJI) is a devastating complication after arthroplasty. Prompt establishment of an infection diagnosis is critical but can be very challenging at present. In order to evaluate the diagnostic accuracy of alpha-defensin or leukocyte esterase for PJI, we performed systematic research in PubMed, Embase, and Cochrane Library to retrieve relevant studies. Data extraction and quality assessment were performed by two reviewers independently. A total of thirty-one eligible studies were finally included in the quantitative analysis. The pooled sensitivity and specificity of alpha-defensin (21 studies) for the diagnosis of PJI were 0.89 (95% confidence interval (CI), 0.83 to 0.93) and 0.96 (95% CI, 0.95 to 0.97), respectively. The value of the pooled diagnostic odds ratios (DOR) of alpha-defensin for PJI was 209.14 (95% CI, 97.31 to 449.50), and the area under the curve (AUC) was 0.98 (95% CI, 0.96 to 0.99). The pooled sensitivity and specificity of leukocyte esterase (17 studies) for the diagnosis of PJI were 0.90 (95% CI, 0.84 to 0.95) and 0.96 (95% CI, 0.93 to 0.97), respectively. The value of the DOR of leukocyte esterase for PJI was 203.23 (95% CI, 96.14 to 429.61), and the AUC was 0.98 (95% CI, 0.96 to 0.99). Based on the results of our meta-analysis, we can conclude that alpha-defensin and leukocyte esterase are valuable synovial fluid markers for identifying PJI with comparable high diagnostic accuracy.

## 1. Introduction

Periprosthetic joint infection (PJI) occurs in more than 2% of patients undergoing joint arthroplasty, acting as the leading cause of total knee arthroplasty failure and the third most common indication for hip revision [1–3]. Meanwhile, PJI imposes a heavy financial burden on patients and the healthcare system. The cost of using a debridement-and-retention protocol and one-stage revision to treat a single PJI is approximately 3-fold and 3.4-fold the cost of primary implantation, respectively [4]. There are some methods in use, such as perioperative antibiotics, antibiotic-impregnated bone cement, and antimicrobial-loaded implants, which can reduce the incidence of PJI but cannot completely eliminate it [5]. A timely diagnosis of an infection may be helpful in eliminating lesions completely and thus avoiding radical treatments such as one-stage or two-stage revision [6].

The most common clinical manifestations of PJI are pain, induration or edema, wound drainage or effusion, surgical site erythema in the early stage, and sinus in the later stage. Conventionally, the most used diagnostic algorithms for the diagnosis of PJI are peripheral blood tests, imaging examination, and microbiological examination. No single test is accurate enough for PJI diagnosis, and the test results must be combined with clinical history and symptoms; however, symptoms of PJI are usually nonspecific in the early stage. Groups such as the American Academy of Orthopedic Surgeons (AAOS), Infectious Diseases Society of America (IDSA), and the Musculoskeletal Infection Society (MSIS) have proposed several criteria for the diagnosis of PJI [7–9], but applications of these definitions are limited in daily clinical practice due to their complexity. A more specific and sensitive routine test for the diagnosis of PJI is therefore badly needed.

Alpha-defensin and leukocyte esterase are both secreted by activated neutrophils in the presence of pathogenic infection. As an antimicrobial peptide, alpha-defensin binds to and destroys invading pathogens [10] while leukocyte esterase has been widely used as an indicator for the assessment of urinary tract infections in the clinic [11]. The content of alpha-defensin is normally detected by lateral flow test [12] or laboratory-based alpha-defensin immunoassay [13] and that of leukocyte esterase by colorimetric strip test [14]. Synovial fluid alpha-defensin and leukocyte esterase were first proposed as novel diagnostic biomarkers for PJI, respectively, by Deirmengian et al. in 2014 [15] and Parvizi et al. in 2011 [16], but their diagnostic efficacy has yet to be confirmed.

In 2016, Wyatt et al. [17] conducted a meta-analysis of the usefulness of alpha-defensin and leukocyte esterase as diagnostic tools for PJI, with pooled sensitivity and specificity being 1.00 (95% confidence interval (CI), 0.82 to 1.00) and 0.96 (95% CI, 0.89 to 0.99), respectively, for alpha-defensin, and 0.81 (95% CI, 0.49 to 0.95) and 0.97 (95% CI, 0.82 to 0.99), respectively, for leukocyte esterase. However, in this systematic analysis [17], only 11 studies were included and four of six studies concerning alpha-defensin came from the same research group, which might hinder the generalization of their results. Additionally, the value of pooled sensitivity of leukocyte esterase was quite different from that reported in the literature published in recent years. Moreover, heterogeneity tests and subgroup analysis were not conducted by Wyatt et al. [17]. We carried out this updated meta-analysis to further assess the diagnostic accuracy of alpha-defensin and leukocyte esterase for periprosthetic joint infection.

## 2. Materials and Methods

The methodological approach to evidence searching and synthesis described in this article was based on the Cochrane Collaboration's diagnostic test accuracy method [18]. We carried out the current systematic review and reported the findings in accordance with the standards of the Preferred Reporting Items for Systematic Reviews and Meta-Analyses (PRISMA) [19]. No ethical approval or informed consent was required for this article because all data were retrieved from published literature. Searching for studies, identification of eligibility, data extraction, and quality assessment were performed by two investigators (ZZ Li and QY Zhang) independently. Any disagreement was resolved through discussion, and the two researchers had to come to a consensus.

### 2.1. Search Strategy

Three electronic databases PubMed, Embase, and Cochrane Library were searched on June 18, 2020, and no time limitation was applied. Vocabulary and syntax were specifically adapted according to the database. We used “periprosthetic joint infection” as our diagnosis of interest and “leukocyte esterase” or “alpha defensin” as our target index, and the full search strategy is shown in Table 1. No language limitation was applied. Reference lists of relevant articles were also screened manually for any additional possible records.

### 2.2. Inclusion Criteria

Studies included in the systematic review needed to meet the following criteria: (1) patients who had undergone joint replacement, (2) sufficient synovial fluid had been aspirated to meet the needs of the tests, (3) the leukocyte esterase or alpha-defensin tests were performed on the synovial fluid, (4) the diagnosis of PJI was confirmed by the MSIS, AAOS, and IDSA guidelines or utilizing a combination of clinical data, which must include microbiological examination, (5) either a prospective or a retrospective study design, and (6) sufficient data could be extracted to construct a 2 × 2 contingency table. If more than one study provided overlapping data, only the most comprehensive or latest one was included. Case reports, commentaries, expert opinion, and narrative reviews were excluded.

### 2.3. Data Extraction and Quality Assessment

Requisite data extracted and recorded to standardized excel files included surname of the first author, publication year, study inclusion interval, country, study design, demographic information of participants, number of infected/total joints, site of arthroplasty, cut-off value, method used to assess alpha-defensin and leukocyte esterase, standard reference, and number of false/true positive and false/true negative cases. The methodological quality of included studies was appraised according to the QUADAS- (Quality Assessment of Diagnostic Accuracy Studies-) 2 tool, which consists of four key domains (i.e., patient selection, index test, reference standard, and flow and timing). Risk of bias was assessed in each domain, and concerns about applicability were assessed in the first three domains with signaling questions. These questions were answered with “yes” for a low risk of bias/concerns, “no” for a high risk of bias/concerns, or “unclear” when relevant information was not clearly provided [20].

### 2.4. Statistical Analyses

Pooled sensitivity, specificity, positive likelihood ratio (PLR), negative likelihood ratio (NLR), and diagnostic odds ratio (DOR) were calculated using the bivariate meta-analysis framework. The bivariate model employs a random-effects approach, and the statistical properties of the bivariate model are suited to performing diagnostic meta-analyses. In addition, summarized receiver operating characteristic (sROC) curves were constructed, with the area under the curve (AUC) depicting the accuracy of tests. Heterogeneity among included studies was assessed using the *I*^2^ statistic. An *I*^2^ value of 0% implied no observed heterogeneity, and values of >50% indicated substantial heterogeneity. For studies with substantial heterogeneity, the Spearman correlation coefficient was calculated to determine whether a threshold effect existed and we also performed metaregression analyses to find the source of variability. Subgroup analyses based on covariates were also performed to ascertain the stability of results.

The value of a two-sided *P* < 0.05 was considered statistically significant in all statistical tests. Stata version 13 (StataCorp, College Station, TX, USA) was used to analyze data from the included studies, and Review Manager Software version 5.3 (Cochrane Collaboration, Oxford, UK) was used to assess the methodological quality of included studies.

## 3. Results

### 3.1. Search Results and Study Selection

**Figure 1:**
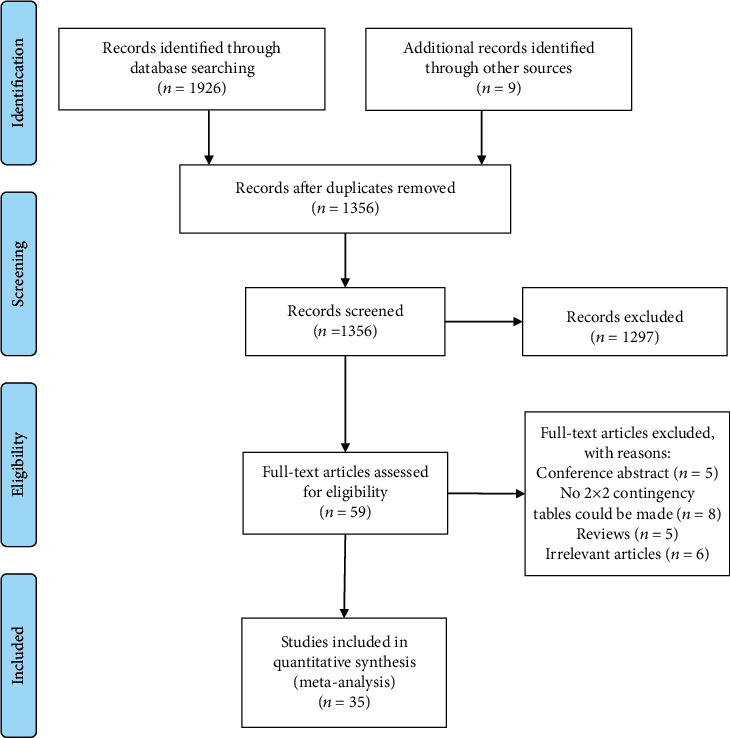
Selection process of included studies.

A total of 1356 records were identified by searching databases and removing duplicates. After the initial screening of titles and abstracts, 59 articles were further assessed by scrutinizing the full texts against the predesigned criteria, and eventually, 35 articles [12, 14–16, 21–51] were included in quantitative analysis. Selection processes for eligible studies are depicted in Figure 1.

### 3.2. Study Characteristics

Twenty-three studies [12, 16, 21, 22, 25, 27–39, 41–44, 47] were prospective, and twelve studies [14, 15, 23, 24, 26, 40, 45, 46, 48–51] were retrospective. Thirty-four studies [12, 15, 16, 21–51] were cohort studies whereas only one study [14] was a case-control study. Twenty-one studies involving a total of 1928 patients (650 joints with PJI) explored the diagnostic accuracy of alpha-defensin [12, 14, 15, 23–27, 29, 30, 33–35, 37, 38, 41, 45, 46, 48–50], among which eight studies [12, 27, 29, 30, 34, 35, 37, 48] used a lateral flow test to assess alpha-defensin, twelve [14, 15, 24–26, 33, 38, 41, 45, 46, 49, 50] adopted a laboratory-based alpha-defensin immunoassay, and the testing methods used were not reported in one studies [23]. The mean ages of included patients ranged from 61.7 to 71.0 years, and the proportion of males ranged from 40.3% to 61.5%.

Meanwhile, seventeen studies involving a total of 1963 patients (571 joints with PJI) explored the diagnostic accuracy of leukocyte esterase for PJI [14, 16, 21, 22, 26, 28, 31, 32, 36, 39, 40, 42–44, 47, 50, 51]. All studies used the standard chemical test strip, among which two studies [16, 32] used an automated reader to define the final result while thirteen studies [14, 21, 22, 26, 28, 31, 36, 39, 40, 42–44, 47, 50, 51] used the naked eye. The mean ages of included patients ranged from 60.3 to 71.0 years, and the proportion of males ranged from 21.7% to 60.9%.

Eventually, 38 datasets were available for the appraisal of diagnostic accuracy of alpha-defensin and leukocyte esterase for PJI. The main characteristics of included studies are summarized in Table 2.

### 3.3. Results of Quality Assessment

**Figure 2:**
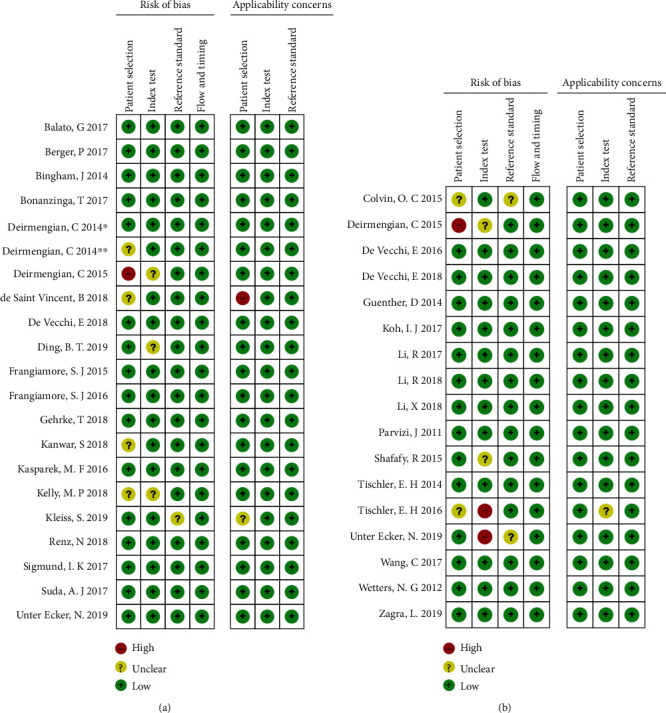
Quality assessment of included studies using QUADAS-2 tool criteria. Red in the figure indicates high risk, yellow represents unclear risk, and green means low risk.

The results of QUADAS-2 assessments for each included study are shown in Figure 2. In each key domain, the proportion of high risk was less than 5%, which indicated that the quality of included studies was good.

### 3.4. Diagnostic Value of Alpha-Defensin for PJI

**Figure 3:**
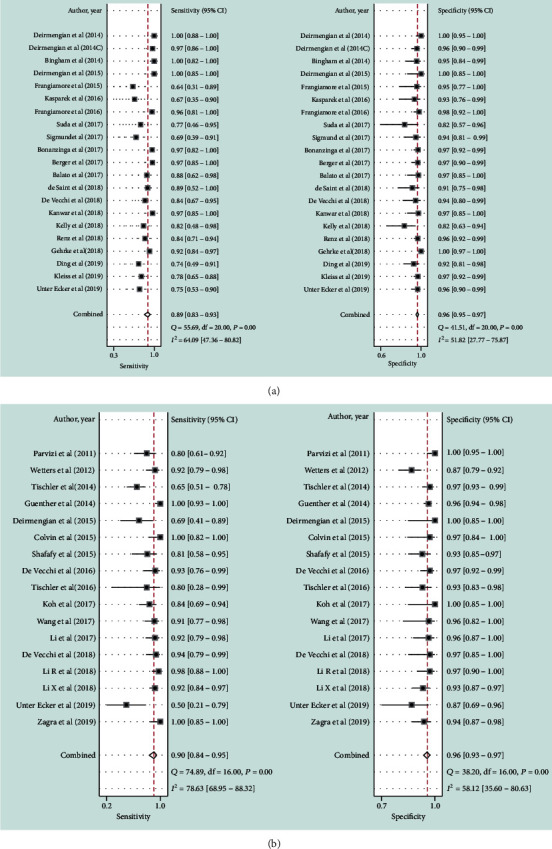
Forest plots of the sensitivity and specificity of alpha-defensin (a) and leukocyte esterase (b) for periprosthetic joint infection across all included studies. Diamonds in the central vertical lines represent pooled sensitivities or specificities with corresponding 95% confidence interval.

**Figure 4:**
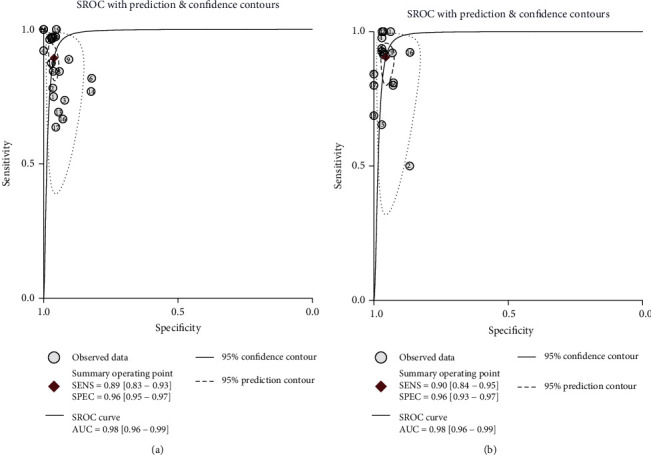
Summarized receiver operating characteristic curve (sROC) of alpha-defensin (a) and leukocyte esterase (b) for periprosthetic joint infection with corresponding 95% confidence region and the 95% prediction region.

**Figure 5:**
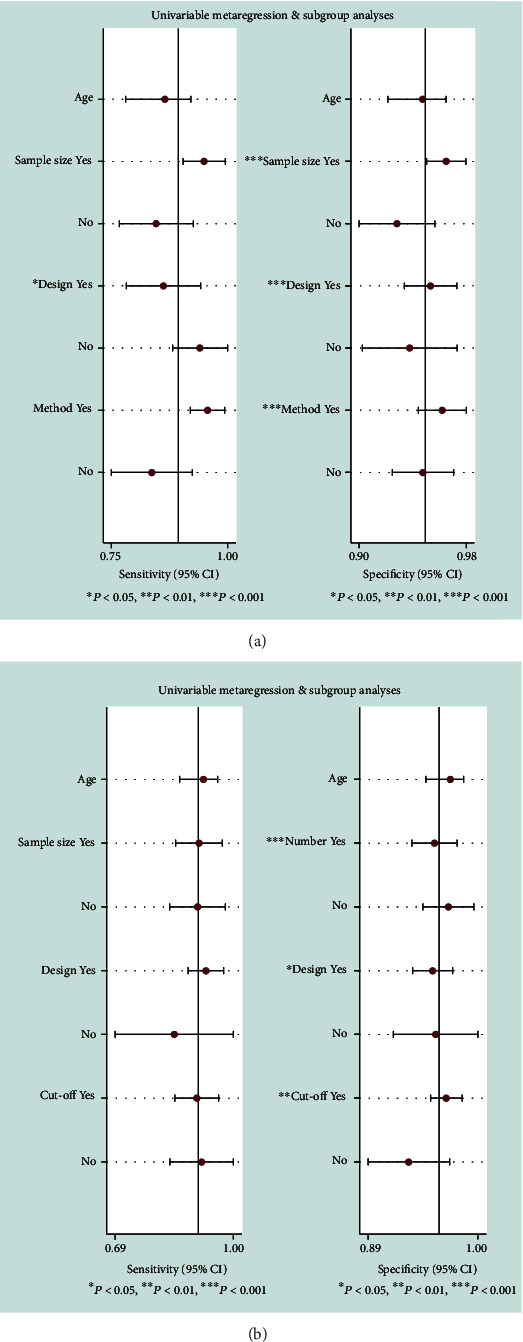
Graphical display of the results of univariable metaregressions of alpha-defensin (a) and leukocyte esterase (b).

As shown in Figure 3, the pooled sensitivity and specificity of alpha-defensin for diagnosing PJI were 0.89 (95% CI, 0.83 to 0.93) and 0.96 (95% CI, 0.95 to 0.97), respectively. The pooled PLR, NLR, and DOR were 23.18 (95% CI, 15.79 to 34.03), 0.11 (95% CI, 0.07 to 0.18), and 209.14 (95% CI, 97.31 to 449.50), respectively. The AUC of alpha-defensin for PJI was 0.98 (95% CI, 0.96 to 0.99) (Figure 4). The *I*^2^ statistics for sensitivity and specificity values were 64.09% (95% CI, 47.36% to 80.82%) and 51.82% (95% CI, 27.77% to 75.87%), respectively, which indicated substantial heterogeneity among included studies. We subsequently performed subgroup and metaregression analysis to explore the source of heterogeneity. The summary data of alpha-defensin for PJI calculated using STATA and estimation of the Spearman correlation coefficient (*P* value < 0.01) indicated that the proportion of heterogeneity was likely due to the threshold effect. In addition, for the nonthreshold effect, we also performed metaregression analysis. Age, patient sample size, study design, and method of testing were used as covariates. The results of metaregression analysis (Figure 5) revealed that patient sample size and method of testing accounted for the heterogeneity of sensitivity and specificity while study design accounted for the heterogeneity of specificity.

Subgroup analyses were performed according to the testing method (lateral flow test or laboratory-based alpha-defensin immunoassay), sample size (<100 or ≥100), and study design (retrospective or prospective), and the pooled results are presented in Table 3.

### 3.5. Diagnostic Value of Leukocyte Esterase for PJI

As shown in Figure 3, the pooled sensitivity and specificity of leukocyte esterase for diagnosing PJI were 0.90 (95% CI, 0.84 to 0.95) and 0.96 (95% CI, 0.93 to 0.97), respectively. The pooled PLR, NLR, and DOR were 20.25 (95% CI, 13.71 to 29.90), 0.10 (95% CI, 0.06 to 0.18), and 203.23 (95% CI, 96.14 to 429.61), respectively. The AUC of leukocyte esterase for PJI was 0.98 (95% CI, 0.96 to 0.99) (Figure 4). The *I*^2^ statistics for sensitivity and specificity values were 78.63% (95% CI, 68.95% to 88.32%) and 58.12% (95% CI, 35.60% to 80.63%), respectively, which indicated substantial heterogeneity among the included studies. The summary data of leukocyte esterase for PJI calculated using STATA and estimation of the Spearman correlation coefficient (*P* value > 0.05) indicated the absence of a threshold effect. In addition, for the nonthreshold effect, we also performed metaregression analysis with age, patient sample size, study design, and cut-off used as covariates. The results of metaregression analysis (Figure 5) revealed that patient sample size, study design, and cut-off accounted for the heterogeneity of specificity.

Among the 17 studies, subgroups were divided according to cut-off value (++ or ++/+) and patient sample size (<100 or ≥100), and the pooled results are presented in Table 3.

## 4. Discussion

Accurate and fast diagnosis of periprosthetic infections remains a challenging problem. The present “gold standard” definition for PJI proposed by the Musculoskeletal Infection Society (MSIS) is adopted by most physicians, which requires accordance with either of two major criteria (sinus tract communication with the prosthesis or a pathogen isolated by culture from at least two separate tissue or fluid samples) or four of six minor criteria (elevated erythrocyte sedimentation rate/ESR, C-reactive protein/CRP, white blood cell/WBC count, and percentage of polymorphonuclear leukocytes/PMN; the presence of purulence; isolation of a microorganism in one culture; and greater than five neutrophils per high-power field) [9]. Our results revealed that alpha-defensin and leukocyte esterase were highly sensitive and specific in identifying PJI (pooled sensitivity of 0.89 and 0.90, respectively, with pooled specificity of 0.96 and 0.96, respectively). We also estimated alpha-defensin and leukocyte esterase combined in the diagnosis of PJI. The corresponding sensitivity and specificity of alpha-defensin associated with leukocyte esterase test's parallel and serial test were 0.989, 0.9216 and 0.801, 0.9984, respectively. The values of AUC for alpha-defensin and leukocyte esterase were both 0.98 (0.96-0.99), indicating a comparable, extremely high diagnostic ability to identify PJI using these two biomarkers.

Alpha-defensin can be detected by lateral flow test or laboratory-based alpha-defensin immunoassay. Based on the results of subgroup analysis, the diagnostic accuracy for PJI using laboratory-based alpha-defensin immunoassay was higher than that using a lateral flow test. Despite being less accurate, the lateral flow test is valuable for its high specificity, portable operation, and short operative time (responses within ten minutes). This method could be an alternative format in rapidly ruling in and, most importantly, ruling out a suspected PJI during surgery [27]. There were two studies that investigated the application of alpha-defensin [41, 50] and one that evaluated leukocyte esterase [50] in diagnosing infection around shoulder prostheses. These reported sensitivity of 63%, 75%, and 50%, respectively, which was lower than other included studies. This might indicate that alpha-defensin and leukocyte esterase were less accurate in diagnosing PJI of the shoulder than that of the hip and knee; however, this conclusion should be interpreted carefully and further studies are warranted. The diagnostic accuracy of alpha-defensin seemed to be slightly better in the retrospective setting and with a larger sample size from our results of subgroup analyses. We further performed univariate metaregression analysis based on method, patient sample size, and study design and found that all factors were responsible for heterogeneity. In addition, threshold effect also accounted for the heterogeneity of studies on alpha-defensin for PJI.

Leukocyte esterase can be detected by a standard chemical test strip. Since Parvizi et al. [16] first proved that the leukocyte esterase strip could be used in the diagnosis of PJI, a standard cut-off value has remained undetermined. Based on the results of subgroup analysis, the diagnostic accuracy for PJI of the leukocyte esterase test with a “++” cut-off was much higher than that with a “++/+” cut-off. Therefore, we suggest that the optimal cut-off value of leukocyte esterase in future studies or clinical applications should be “++.” In addition, the subjectivity of interpretation of the result of the leukocyte esterase strip might cause a possible bias. Two studies [16, 32] used an automated reader instead of using the naked eye to define the final result. The sensitivities of these two studies (sensitivity = 0.80 and 0.84, respectively) were far below the pooled sensitivity of the current meta-analysis while the specificities of these two studies (specificity = 1.00) were far above the pooled specificity. We cannot conclude that it might be more accurate if the colorimetric analysis of the strip is performed by an automated reader, and further studies are warranted to evaluate the accuracy of an automated reader to define the final result of the test strip. In addition, the diagnostic accuracy of leukocyte esterase seemed to be slightly better in the prospective setting from our results of subgroup analyses. We further performed univariate metaregression analysis for cut-off, patient sample size, and study design and found that all factors were responsible for heterogeneity.

The test for alpha-defensin is simple, standardized, and validated to provide uniform results for all surgeons in clinical practice. The laboratory-based alpha-defensin immunoassay has been demonstrated to have the highest accuracy ever reported but requires more time for a response [52]. The novel lateral flow device is an alternative handy format, which is easy to use but shows lower sensitivity [30]. Meanwhile, the routine detection of alpha-defensin is much more expensive ($760 per test) than that for leukocyte esterase ($0.17 per test). The advantage of leukocyte esterase for PJI diagnosis is that the method of testing is convenient and quick, returning a highly accurate result within one to two minutes, but the disadvantage is that subjective opinions might exist and samples contaminated with blood might interfere with the reading result [28]. When a patient shows any signs of infection after total knee or hip arthroplasty, such as fever, swelling, or an elevated level of ESR or CRP, it is necessary to confirm a diagnosis of PJI and regardless of age, the leukocyte esterase test or alpha-defensin test should be carried out for both men and women. Both alpha-defensin and leukocyte esterase are excellent synovial fluid markers with comparable accuracy; the leukocyte esterase test is recommended as the first resort, because the alpha-defensin test is more expensive.

The strengths of the current study lie in the following two aspects. First, 31 articles were included and to our knowledge, this is the largest meta-analysis on this topic. Additionally, subgroup and metaregression analyses were conducted, which enabled us to analyze the extracted data from multiple perspectives and investigate the source of heterogeneity among the included studies.

Potential limitations of this meta-analysis should also be considered. First, three studies of alpha-defensin [14, 15, 45] were reported by the same research group which instructed a commercial company to perform the alpha-defensin test; there might therefore have been a conflict of interest. In addition, sensitivities of these three studies [14, 15, 45] were evidently higher than those of the other included studies, which might be the reason why the pooled sensitivity of Wyatt's meta-analysis was much higher than that in our study.

## 5. Conclusions

Based on the results of the current meta-analysis, we conclude that alpha-defensin and leukocyte esterase are both excellent synovial fluid markers for diagnosing periprosthetic joint infection, with comparable and extremely high accuracy. Detection of alpha-defensin and leukocyte esterase are very convenient and could be performed preoperatively or intraoperatively.

## Figures and Tables

**Table 1:** Search strategy.

Alpha-defensin search	Leukocyte esterase search
(1) Alpha-defensins or alpha defensins (2) Neutrophil antimicrobial peptides or antimicrobial peptides, neutrophil or peptides, neutrophil antimicrobial (3) Arthroplasty or joint prosthesis or joint replacement (4) Periprosthetic infection or prosthetic joint infection or prosthesis-related infections (5) Bacterial infections or surgical wound infection (6) 1 or 2 (7) 4 or 5 (8) 3 and 7 (9) 6 and 8	(1) Leukocyte esterase or leucocyte esterase (2) Leukocyte indoxyl esterase or indoxyl esterase (3) Arthroplasty or joint prosthesis or joint replacement (4) Periprosthetic infection or prosthetic joint infection or prosthesis-related infections (5) Bacterial infections or surgical wound infection (6) 1 or 2 (7) 4 or 5 (8) 3 and 7 (9) 6 and 8

Database: PubMed, Embase, and Cochrane Library. Other databases: subject-specific databases, dissertation databases, or grey literature databases.

**Table 2:** Characteristics of the included studies.

Study (published year)	Inclusion interval	Country	Infected/total joints	Male/female	Mean age (y) (range)	Study design/type	Site of arthroplasty	Method of testing	Cut-off value	Standard reference	Biomarkers of interest
Ding et al., 2019 [48]	2015-2018	Singapore	19/51	NA	67	R Cohort	NA	Lateral flow	Cassette	MSIS	AD
Kleiss et al., 2019 [49]	2015-2018	Germany	55/202	93/109	NA	R Cohort	Knee: 90 Hip: 112	ELISA	5.2 mg/L	MSIS	AD
Unter Ecker et al., 2019 [50]	2015-2018	Germany	24/105	38/67	66 (36-88)	R Cohort	Shoulder	ELISA Chemstrip 7	5.2 mg/L ++	MSIS	AD and LE
Gehrke et al., 2018 [25]	2015-2016	Germany	76/195	77/114	NA	P Cohort	Knee: 99 Hip: 96	Lateral flow and ELISA	Cassette/1.0 S/Co^∗^	MSIS	AD
Renz et al., 2018 [12]	2016-2017	Germany	45/212	106/106	70 (41-94)	P Cohort	Knee: 151 Hip: 61	Lateral flow	Cassette	MSIS	AD
Kelly et al., 2018 [23]	2015-2017	USA	11/39	NA	63 (33-88)	R Cohort	Knee: 33 Hip: 6	Sent to the CD Diagnostics^#^	NA	MSIS	AD
Kanwar et al., 2018 [24]	2014-2016	USA	35/70	NA	66	R Cohort	Knee and hip	ELISA	5.2 mg/L	MSIS	AD
De Vecchi et al., 2018 [26]	2015-2017	Italy	32/66	37/29	68 (63-73)	R Cohort	Knee: 45 Hip: 21	ELISA Chemstrip 7	5.2 mg/L ++/+	MSIS^c^	AD and LE
de Saint et al., 2018 [27]	2015-2017	France	9/41	24/15	35-87	P Cohort	Sites^b^	Lateral flow	Cassette	MSIS	AD
Balato et al., 2017 [35]	2015-2016	Italy	16/51	23/28	63 (48-39)	P Cohort	Knee	Lateral flow	Cassette	MSIS	AD
Berger et al., 2017 [34]	2015-2016	Belgium	34/121	NA	63.5 (36-88)	P Cohort	Knee: 85 Hip: 36	Lateral flow	Cassette	MSIS	AD
Bonanzinga et al., 2017 [33]	2015.04-2015.10	Germany	29/156	66/90	NA	P Cohort	Knee: 65 Hip: 91	CD Labs kit^##^	1.0 S/Co^a^	MSIS	AD
Sigmund et al., 2017 [30]	2015.01-2015.10	Austria	13/49	28/22	65 (20-89)	P Cohort	Knee: 17 Hip: 30 Elbow: 1 Shoulder: 1	Lateral flow	Cassette	MSIS	AD
Suda et al., 2017 [29]	2016.04-2016.06	Germany	13/30	17/11	67.7 (39-88)	P Cohort	Knee: 19 Hip: 11	Lateral flow	Cassette	MSIS	AD
Frangiamore et al., 2016 [38]	2013-2014	USA	27/116	49/53	63.3 ± 11.9	P Cohort	Knee and hip	ELISA	5.2 mg/L	MSIS	AD
Kasparek et al., 2016 [37]	2014-2015	Austria	12/40	NA	71 (41-91)	P Cohort	Knee: 29 Hip: 11	Lateral flow	Cassette	MSIS	AD
Frangiamore et al., 2015 [41]	2012-2013	USA	11/33	13/17	61.7 ± 12.4	P Cohort	Shoulder	ELISA	NA	Own criteria	AD
Deirmengian et al., 2015 [14]	2012.01-2012.08	USA	23/46	28/18	63/67	R Case-control	Knee: 43 Hip: 3	ELISA Chemstrip 7	5.2 mg/L ++(+)	MSIS	AD and LE
Bingham et al., 2014 [46]	2013.01-2013.06	USA	19/61	NA	NA	R Cohort	NA	CD Labs kit^##^	7.72 mg/L	MSIS	AD
Deirmengian et al., 2014 [45]	NA	USA	37/149	70/79	65 (41-89)	R Cohort	Knee: 116 Hip: 33	ELISA	5.2 mg/L	MSIS	AD
Deirmengian et al., 2014 [15]	NA	USA	29/95	44/51	67 (41-89)	R Cohort	Knee: 84 Hip: 11	ELISA	4.8 mg/L	MSIS	AD
Zagra et al., 2019 [51]	2015-2017	Italy	23/119	57/62	66.6 (35-84)	R Cohort	Hip	Chemstrip 7 urine test strips	++/+	MSIS	LE
Li et al., 2018 [21]	2014-2016	China	88/204	81/117	63	P Cohort	Knee: 125 Hip: 79	Aution Sticks 10PA, Japan	++(+)^g^	MSIS	LE
Li et al., 2018 [22]	2016-2017	China	43/110^d^	54/79	65 (18-87)	P Cohort	Knee and hip	Aution Sticks 10PA, Japan	++	MSIS	LE
Li et al., 2017 [31]	2015.01-2015.12	China	38/93	34/59	61.89 (22-89)	P Cohort	Knee: 29 Hip: 64	Aution Sticks 10PA, Japan	++	MSIS	LE
Wang et al., 2017 [28]	2014-2015	China	35/63^e^	34/37	60.3	P Cohort	Knee: 49 Hip: 23	Combur10 and Aution Sticks	++	MSIS	LE
Koh et al., 2017 [32]	2013-2015	Korea	38/60	13/47	71 (50-85)	P Cohort	Knee	3 different types^f^	++(+)^g^	MSIS	LE
Tischler et al., 2016 [36]	2010-2015	USA	5/61	30/31	64.1 (45-80)	P Cohort	Hip	NA	++	MSIS	LE
de Vecchi et al., 2016 [39]	2014-2015	Italy	27/129	66/63	64 (17-88)	P Cohort	Knee: 84 Hip: 45	Dirui Industrial	++/+	MSIS	LE
Shafafy et al., 2015 [40]	2012-2013	UK	21/103^d^	40/54	NA	R Cohort	Knee: 79 Hip: 26	Multistix 8 SG	++/+	IDSA	LE
Colvin et al., 2015 [42]	2013-2014	USA	19/52	NA	69.1 (31-91)	P Cohort	Knee, hip, and elbow	Chemstrip 10 or Chemstrip 7	++	AAOS	LE
Guenther et al., 2014 [44]	NA	Germany	50/354	NA	67 (56-78)	P Cohort	Knee, hip, and shoulder	Roche Diagnostics	++	MSIS	LE
Tischler et al., 2014 [43]	2009-2013	USA	52/189	90/99	63 (21-90)	P Cohort	Knee: 154 Hip: 35	Chemstrip 7 urine test strips	++(+)^g^	MSIS	LE
Wetters et al., 2012 [47]	NA	USA	39/158^d^	97/126	63.3 (33-38)	P Cohort	Knee and hip	Chemstrip 7 urine test strips	++/+	MSIS	LE
Parvizi et al., 2011 [16]	2007-2010	USA	30/108	48/60	64 (28-89)	P Cohort	Knee	Chemstrip 7 urine test strips	++(+)^g^	MSIS	LE

^#^A company provides commercial alpha-defensin immunoassay. ^##^The test kits provided by CD Diagnostics was a laboratory-based *α*-defensin immunoassay. ^a^1.0 S/Co was identified as 5.2 mg/L^−1^ in Deirmengian et al. [45]. ^b^There were 23 that had total joint prostheses, 13 total hip prostheses, and 3 total femoral replacements, and then 42 tests were performed. ^c^The study used the definition of the International Consensus Meeting of Philadelphia but excluding elevated synovial fluid count or positive leukocyte esterase as a minor criterion. ^d^Some patients had samples excluded because of the color disturbance caused by blood contamination. ^e^Nine of 72 aspirations were excluded due to the lack of synovial fluid. ^f^Three different types including Aution Eleven, Arkray, Kyoto, Japan; Clinitek500, Siemens, Munich, Germany; and Urisys 2400, Roche Diagnostics, Mannheim, Germany. ^g^Both ++ and ++/+ were used as cut-off value. NA = not available; ELISA = Enzyme-Linked ImmunoSorbent Assay; AD = alpha-defensin; LE = leukocyte esterase; P = prospective; R = retrospective; MSIS = Musculoskeletal Infection Society; IDSA = Infectious Diseases Society of America; AAOS = American Academy of Orthopedic Surgeons.

**(a):** 

Bivariate model analysis							
Sen (95% CI)	*I* ^2^_sen_ (95% CI)	Spe (95% CI)	*I* ^2^_spe_ (95% CI)	PLR (95% CI)	NLR (95% CI)	DOR (95% CI)	AUC (95% CI)
Alpha-defensin							
0.89 (0.83-0.93)	64.09 (47.36-80.82)	0.96 (0.95-0.97)	51.82 (27.77-75.87)	23.18 (15.79-34.03)	0.11 (0.07-0.18)	209.14 (97.31-449.50)	0.98 (0.96-0.99)
Leukocyte esterase							
0.90 (0.84-0.95)	78.63 (68.95-88.32)	0.96 (0.93-0.97)	58.12 (35.60-80.63)	20.25 (13.71-29.90)	0.10 (0.06-0.18)	203.23 (96.14-429.61)	0.98 (0.96-0.99)

**(b):** 

Subgroup analyses							
Subgroup parameter	Sen (95% CI)	*I* ^2^_spe_ (95% CI)	*P* value	Spe (95% CI)	*I* ^2^_spe_ (95% CI)	*P* value	DOR (95% CI)
Alpha-defensin							
Method							
Lateral flow	0.82 (0.73-0.89)	33.91 (0.00-87.72)	0.16^∗^	0.94 (0.91-0.96)	18.99 (0.00-79.61)	0.28^∗^	79.72 (34.85-182.36)
ELISA or CD Labs kit	0.95 (0.88-0.98)	75.41 (60.90-89.92)	<0.001	0.97 (0.96-0.98)	27.10 (0.00-78.48)	0.19^∗^	665.36c (227.02-1950)
Patient sample size							
<100	0.89 (0.78-0.95)	64.28 (43.09-85.47)	<0.001	0.95 (0.91-0.97)	51.23 (20.29-82.16)	0.02^∗^	141.23 (42.54-468.84)
>100	0.91 (0.84-0.95)	68.56 (45.32-91.80)	<0.001	0.97 (0.96-0.98)	0.00 (0.00-100.00)	0.57^∗^	336.68 (148.54-763.10)
Study design							
Prospective	0.86 (0.77-0.92)	56.76 (26.26-87.27)	0.01	0.95 (0.93-0.97)	20.56 (0.00-76.82)	0.25^∗^	133.34 (53.32-333.43)
Retrospective	0.93 (0.82-0.98)	75.83 (60.91-90.76)	<0.001	0.96 (0.93-0.98)	68.45 (47.65-89.25)	<0.001	337.72 (77.59-1469.60)
Leukocyte esterase							
Cut-off value							
++	0.89 (0.79-0.95)	81.75 (72.24-91.26)	<0.001	0.96 (0.94-0.97)	38.01 (0.00-80.27)	0.09^∗^	202.37 (80.55-508.41)
++/+	0.92 (0.87-0.96)	69.00 (47.50-90.49)	<0.001	0.89 (0.83-0.93)	79.06 (65.87-92.24)	<0.001	102.20 (43.64-239.34)
Patient sample size							
<100	0.87 (0.75-0.93)	70.89 (49.73-92.04)	<0.001	0.96 (0.92-0.98)	35.05 (0.00-88.02)	0.15^∗^	142.87 (43.09-473.72)
>100	0.93 (0.83-0.97)	84.22 (75.00-93.44)	<0.001	0.95 (0.93-0.97)	72.42 (53.82-91.02)	<0.001	267.26 (99.77-715.92)
Study design							
Prospective	0.92 (0.85-0.96)	78.81 (67.32-90.30)	<0.001	0.96 (0.94-0.98)	67.25 (47.42-87.08)	<0.001	276.70 (128.79-594.49)
Retrospective	0.85 (0.62-0.95)	79.00 (60.79-97.22)	<0.001	0.93 (0.89-0.96)	17.08 (0.00-100.00)	0.31^∗^	80.18 (18.07-355.81)

Sen = sensitivity; Spe = specificity; PLR = positive likelihood ratio; NLR = negative likelihood ratio; DOR = diagnostic odds ratios; AUC = area under curve. ^∗^The results of *P* value are larger than 0.05 which indicates no observed heterogeneity.

## Data Availability

The datasets used and/or analyzed during the present study are available from the corresponding author on reasonable request.
